# Predispose, precipitate, perpetuate, and protect: how diet and the gut influence mental health in emerging adulthood

**DOI:** 10.3389/fnut.2024.1339269

**Published:** 2024-03-05

**Authors:** Michael Warren, Colleen O’Connor, Ju Eun Lee, Jeremy Burton, David Walton, Justine Keathley, Michael Wammes, Elizabeth Osuch

**Affiliations:** ^1^Department of Psychiatry, Schulich School of Medicine and Dentistry, University of Western Ontario, London, ON, Canada; ^2^School of Food and Nutritional Sciences, Brescia University College, London, ON, Canada; ^3^Geriatrics, Department of Psychiatry, University of Toronto, Toronto, ON, Canada; ^4^Department of Surgery, Microbiology and Immunology, Lawson Health Research Institute, Western University, London, ON, Canada; ^5^School of Physical Therapy, Schulich School of Medicine and Dentistry, Western University, London, ON, Canada; ^6^Department of Human Health and Nutritional Sciences, College of Biological Science, University of Guelph, Guelph, ON, Canada; ^7^London Health Sciences Centre, Lawson Health Research Institute, London, ON, Canada; ^8^First Episode Mood and Anxiety Program, London Health Sciences Centre, London, ON, Canada

**Keywords:** gut-brain microbiome axis, psychiatry, nutrition, clinical formulation, preventive psychiatry, emerging adult

## Abstract

Medicine often employs the 4Ps of *predisposing, precipitating, perpetuating*, and *protective* factors to identify salient influences on illness states, and to help guide patient care. Mental illness is a significant cause of morbidity and mortality worldwide. Mental health is a complex combination of biological, psychological, environmental, and social factors. There is growing interest in the gut-brain-microbiome (GBM) axis and its impact on mental health. We use the medical model of the 4Ps to explore factors involving the connection between nutrition and the GBM axis and their associated risks with mental health problems in emerging adults (EAs), a life stage when mental illness onset is the most common. We review the impact of current dietary trends on the GBM and on mental health, and the role that gut microbiome-based interventions can have in modulating the GBM axis of EAs. We discuss the implications of gut health on the GBM and areas for clinical intervention.

## Introduction

Mental illness is a significant cause of disability and death worldwide for the general population (1). Among 15–29 year-olds depression is one of the leading causes of disability, and related suicides are the fourth leading cause of death (2). In Canada, mental illnesses and substance use disorders are the second leading cause of all-age years lived with disability (3) and adolescence is the most common age of onset of these illnesses (4). Given the impact of depressive symptoms, for example, on quality of life (5), and system capacity limitations regarding conventional treatment (6), the expansion of evidence-based strategies for prevention and early intervention for mental health problems in emerging adults (EAs; ages 16–25) is greatly needed (7).

Massetti et al. (8) analyzed data from over 90,000 young adults and reported significant associations between chronic mental health disorders and many known risk factors or behaviors associated with other health conditions, including smoking, binge drinking, inadequate sleep, limited leisure time physical activity, limited meaningful leisure time activity and having overweight or obese body habitus (females only) (8). Although this study did not investigate diet or nutrition directly, burgeoning research implicates the gut-brain-microbiota (GBM) axis as an important mechanism to consider in understanding and potentially improving mental health. The GBM axis explains the complex interactions among food/beverages/nutrients/bioactives (i.e., diet), the gut, and brain health, including mental health. Several mechanisms are implicated in regulating the GBM axis, including the hypothalamic–pituitary–adrenal axis (HPA), microbial metabolites, hippocampal neurogenesis, neuronal pathways (via the vagus nerve), neuroactive pathways (serotonin and neuroactive metabolites), the kynurenine pathway, and immune pathways (9). Importantly, recent findings suggest a unique vulnerability in the gut microbiota (GM) during the period of emerging adulthood (10–12). The GM regulates the integrity of the intestinal membrane and inflammation along the gastrointestinal tract (13). Through the GBM axis, alterations in membrane integrity and inflammation appear to have a bidirectional relationship with brain health, mood and behavior (14). Our group has previously raised the role of the gut microbiome in EAs as an area of interest for intervening in the trajectory of mental illnesses (15).

The relationships among the GBM axis, food and medication are complex and multifaceted. This is partly because the GBM also appears to be influenced by many factors such as genetics, diet, age, sex, exercise, sleep, environmental exposures, and substance use (16–18), thereby confounding simple bivariate analyses. Medications, including psychotropic medications used to treat mental illnesses, such as antidepressants or antipsychotics, carry side effects with short- and long-term usage, including effects on the microbiota (19, 20). Unfortunately, concurrent with the positive effect of symptom improvement, psychotropic medications often result in weight gain. Weight gain associated with medications may persist long-term during their use, leading to a lack of adherence to treatment and/or predisposition to chronic metabolic diseases (21).

**Figure 1:**
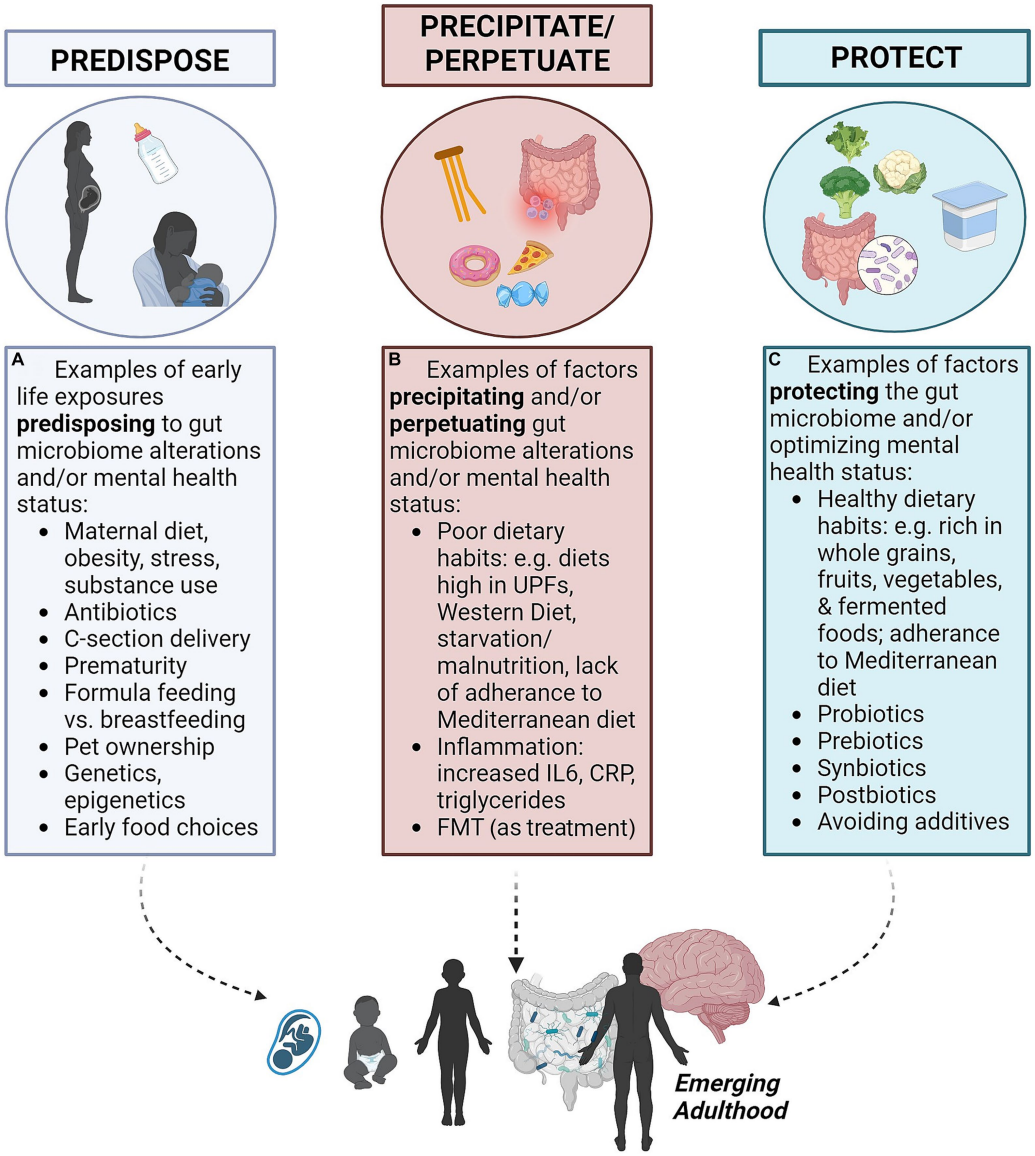
The 4P’s of gut-brain microbiome axis and mental health (created with BioRender.com). These four factors contribute to mental health outcomes for emerging adults via the gut-brain microbiome axis. **(A)** Predisposing factors may occur before birth or early in life. **(B)** Precipitating and perpetuating factors include dietary habits in early life and into adulthood. **(C)** Protective factors may counteract precipitating and perpetuating factors.

The *predisposing, precipitating, perpetuating*, and *protective* factors framework, referred to as the “4Ps,” is used in medicine for organizing contributing factors in a clinical case and to communicate illness and risks with patients (22) (see Table 1). *Predisposing* factors refer to factors that increase the vulnerability of a person to the onset of a disease process, such as genetic inheritance, ongoing environmental exposures and epigenetic modifications (Figure 1A). *Precipitating* factors are described as events contributing to, or triggering, the onset of the disease, such as traumatic events, acute environmental exposures, disease states or illness episodes (Figure 1B). *Perpetuating* factors pertain to ongoing events, behaviors, or stimuli that maintain the course of the disease, such as ongoing traumatic or environmental exposures, dietary intake, medication misuse, the uninterrupted course of the illness itself, or difficulty changing health-compromising habits. The reversal of a perpetuating factor of illness is often a treatment for that illness (Figure 1B). *Protective* factors refer to characteristics that reduce the burden or the severity of the disease and contribute to resilient overall functioning, such as good social support, the absence of predisposing or precipitating factors, regular exercise and the maintenance of other health-promoting habits (22, 23) (Figure 1C). The framework presupposes a biopsychosocial perspective and offers insight and structure to the course of illness and its management, assuming a multifactorial and multidisciplinary approach.

**Table 1:** 4 “P” framework.

Factor	Definition	Examples of contributors
Predisposing	Factors that increase the vulnerability of an individual for the onset of a disease process.	Genetic inheritance or epigenetic modifications. Early life exposure to agents that affect gut microbiota
Precipitating	Factors or events contributing to, or triggering, the onset of the disease.	Traumatic events, disease states or illness episodes. Acute or chronic environmental or dietary exposures that alter gut microbiota
Perpetuating	Ongoing events, behaviors, or stimuli that maintain the course of the disease.	Ongoing traumatic exposures, medication misuse, the uninterrupted course of the illness itself, or difficulty changing health-compromising habits. Ongoing environmental or dietary exposures
Protective	Factors that reduce the burden or the severity of the disease and contribute to overall functioning.	Good social supports, beneficial dietary habits, use of probiotics/prebiotics, regular exercise, and the maintenance of other health-promoting habits

Adapted from Selzer and Ellen (22) and Racine et al. (23).

Since considering mental health as related to the GBM is an emerging scientific field, we propose that a framework to standardize language and scaffold these complex concepts as they relate to clinical care represents an important contribution to meaningfully moving forward. In this review, we demonstrate an application of the 4Ps framework to explore the impact of dietary intake on the GM and how the GBM may function as predisposing, precipitating, perpetuating and protecting factors in the contexts of the mental health of EAs as depicted in Figure 1. This review aims to narratively summarize the current state of knowledge in this emerging field and identify priority gaps regarding understanding interventions of the GBM for adjunct or primary approaches to targeting mental illness.

### Diet and the microbiome as *predisposing* factors for mental illness

Even before the GM colonization of the newborn infant there are influences at play relating to maternal mental health prenatally (*viz.*, *in utero*) (Figure 1A). Maternal factors such as diet, obesity, substance use (24), immune activation, and stress have been found to influence the microbiota composition in both preclinical and clinical models of the newborn (25). Research has demonstrated an association between maternal fecal microbiota during the third trimester of pregnancy (specifically, alpha diversity) and child internalizing behavior at age two, which are common preludes to depression and anxiety (26). GM in early postnatal life is influenced by a variety of factors including mode of delivery, prematurity, prenatal nutrition, pet ownership, geographical location/ethnicity and socioeconomic factors, physical illness, antibiotic use, and maternal stress during pregnancy (25, 27). Breastfeeding, compared to formula feeding, is associated with distinct infant intestinal microbiota (28, 29), though not necessarily early-life mental health outcomes (30). Breastfeeding is associated with a reduced risk of early obesity in young children (31, 32), and better cognitive development (33). Conversely, formula-fed infants whose feeding was matched from a nutrient and calorie perspective have higher associated risks of diseases such as eczema and asthma (34). Additionally, emerging evidence suggests that pre- and probiotics given to infants (e.g., added to formula feeding) and children may help to optimize the gut microbiome (27).

Impactful across the lifetime, studies have implicated exposures *in utero* and from breastmilk to food choices in later life (35). There is abundant evidence that early life feeding experiences influence later childhood and adolescent food choices (36, 37), potentially into adulthood. Because of the association between high fiber diets low in sugars and fats and both better mental and physical health (38), early life feeding styles are a predisposing factor for mental health outcomes. A recent systematic review has concluded that ultra-processed food (UPF) consumption is associated with both depression and anxiety (39), as well as chronic physical ailments. The consumption of fruits and vegetables, conversely, has been associated with a variety of measures of mental wellbeing (40). A varied diet with low levels of UPF intake and high levels of fruits and vegetables is associated with mental wellness (40–42).

There is strong theoretical rationale linking food intake and mental health as being mediated through the GBM (12). The prevalence of a less nutrient-rich and more processed diet with numerous additives may well be predisposing whole segments of the human population to poor mental health, starting at a young age. Exposure to a variety of food-based and environmental factors, including food emulsifiers, compounds that affect the endocrine system, and bio-toxic compounds may also predispose to GBM alterations and mental unwellness (43, 44). It may prove useful for public health initiatives to convey information related to diet and environmental exposures as predisposing factors of mental wellness and illness.

### Diet and the microbiome as *precipitating* factors for mental illness

Food intake and the GBM may also play an important role as a *precipitating* factor for mental illnesses in EAs, made vulnerable by the instability of the GBM in adolescence and young adulthood, a period during which most mental illnesses have their onset (10, 11). Several studies and reviews over the past decade have explored the GBM axis and the complex bidirectional relationship between diet and mental health (14, 45–47), including among adolescents (12). In the context of EAs, this relationship is particularly complex, since eating behaviors, experimentation with substances and hormonal states are often irregular during this developmental stage. Puberty and emerging adulthood are periods of fluctuations of endogenous hormones as well as the maturation of the HPA axis (48). Poor nutritional status and/or alterations of the GM by compounds in the food chain or environment may, therefore, have a unique impact on both the GM and the HPA axes of EAs, precipitating neuropsychiatric symptoms (Figure 1B).

Processed foods and UPFs, as defined by the NOVA classification (49), tend to be elevated in total energy, while providing minimal nutritional benefits (50). The Western Diet (WD) is generally high in processed foods as well as high in saturated fats, omega-6 fatty acids (FAs), refined sugar, salt, preservatives, colorants, artificial flavors, sweeteners, and emulsifiers, and low in fiber (43, 51, 52). Among EAs in Canada, evidence suggests that this represents a dominant proportion of their diet (53).

High-fat diets have been associated with GM dysbiosis (51, 54), and subsequent microbial and metabolite changes have been found to occur with increased consumption of the WD. Increases in members of Firmicutes and Proteobacteria phyla have been noted, and inversely, a proportional reduction in *Bacteroides*, Verrucomicrobia, *Eubacterium rectale*, *Clostridium coccoides*, and *Bifidobacterium* are thought to occur (54). These microbial changes are thought to be associated with a reduction in certain short-chain fatty acids (SCFAs) and an altered gut permeability, leading to subsequent elevated levels of systemic inflammation (54).

The WD has been associated with cognitive impairments and emotional disorders through a variety of proposed mechanisms. Long-term exposure to the WD can induce addictive-like eating behaviors (51). The dysbiosis in the GM induced by the WD further supports its impact on the brain health via the GBM axis (51). This can occur through the induction of proinflammatory cytokine cascades (44), which thereby activate the immune system to cause neuroinflammation, further increasing the risk for emotional disorders (51). The WD and other poor food choices can result in HPA axis hyperactivation, reduced hippocampal neurogenesis, altered kynurenin pathway, mitochondrial dysfunction, reduced serotonin and dopamine levels, and, again, the release of inflammatory cytokines (9, 15) thereby negatively impacting the function of the hippocampus, hypothalamus, amygdala, and higher cortical regions (51).

Within the WD, elevated UPF consumption is associated with systemic inflammation (41). Recent research has found that increased UPF consumption was associated with elevated high sensitivity C-reactive protein levels, a systemic inflammatory maker, among adults (41). Multiple reviews have suggested that elevated systemic and neurological inflammation are associated with psychiatric disorders (51, 54). Dantzer et al. (55) reviewed the role that inflammation and subsequent immune system activation has on depressive symptoms. These authors point out that activation of the immune system through pro-inflammatory cytokines during acute infection induce neurovegetative symptoms in animal models consistent with changes seen in depressive disorders (55). There is also growing evidence, recently summarized by Ortega et al., of microbiota abnormalities associated with bipolar disorders, with neuroinflammation playing a major role (20).

Mounting evidence is beginning to show the negative mental health associations with UPF consumption among adults (41, 56–58). In a cross-sectional study by Hecht et al. (56), associations between UPF consumption and mental illness-related symptoms were investigated. Their findings showed that populations with diets elevated in UPFs tended to report higher anxiety and depression symptoms (56), as well as other non-communicable chronic illnesses (43). A recent systematic review and meta-analysis of cross-sectional, prospective cohort, and case–control studies showed that UPF consumption was associated with an elevated risk of depression (58). These authors further showed, through dose–response analysis, that adult participants had an 11% percent increase in depressive symptoms for each 10% increase in UPF consumption of their total diet (58). Similarly, a cross-sectional study of Mediterranean adults showed that elevated UPF consumption was associated with the presence of depressive symptoms, with results further accentuating the relationship when accounting for adherence to Mediterranean societal eating habits otherwise (57). Godos et al. (57) hypothesized that the association between UPFs and depressive symptoms may be due to underlying neurobiological changes rather than solely nutritional quality. This is generally related to low-grade but persistent inflammation that is associated with UPF consumption (43, 44, 59). The implications of this research are that UPFs may be more directly causative of unhealthy brain function via GM dysbiosis, rather than being harmful via lack of some critical nutrient(s).

Other researchers have suggested a similar and shared mechanism between the role of inflammation with coronary heart disease and depression (60). These authors found that coronary heart disease and depression shared multiple risk factors, including elevations in triglycerides and inflammatory markers, including interleukin 6 (IL-6) and CRP (60). Khandaker et al. (61) conducted a longitudinal study and found that children with elevated IL-6 levels at age 9 were more likely than their peers to develop depression or experience psychotic episodes by age 18 even while accounting for sex, BMI, social class, past psychiatric illness and maternal depression (61). These results further suggest an underlying relationship between inflammation and mental illness. Together, this evidence supports the hypothesis that elevated UPF consumption among children and young adults increases the risk of elevated inflammatory markers, which may precipitate adverse mental health outcomes.

The WD and UPF consumption, as well as exposure to other environmental GM disrupters among young adults almost certainly play a precipitating role in the mental health challenges that have been on the rise in the last decade (43, 62). While the transition period from adolescence through to adulthood is marked with many life changes, evidence suggests that food intake plays a key role in the development of mental illness. The WD, and UPFs in particular, appear to contribute to inflammatory changes and place youth and young adults at increased risk for the precipitation of mental illnesses.

### Diet and the microbiome as *perpetuating* factors for mental illness

Diet, specifically a sub-optimal diet, can play a key part in the maintenance and further perpetuation of mental health symptoms (Figure 1B). One extreme example is the role that malnutrition plays in negative affective symptoms within individuals diagnosed with anorexia nervosa (AN). Depressive symptoms within the context of AN tend to vary in severity across phases of the illness. Previous researchers have suggested that during the underweight stage of illness, depressive and anxiety symptoms may be exacerbated. Indeed, these symptoms demonstrated improvement (but not resolution) after weight restoration due to improved nutritional intake (63). These authors suggest that malnutrition and starvation in the underweight state of AN contribute to worsening mental health symptoms (63). These results imply a cyclical effect, with anxiety and depressive symptoms worsening as AN severity worsens, further impacting functioning through mood/anxiety symptoms that reduces healthy eating. Consistent with this, Pleplé et al. (64) found that AN symptom severity was correlated with anxiety and depressive symptoms during acute hospitalizations; and treatment of nutritional status itself corresponded with improvement in mood and anxiety symptoms (64).

Studies have shown that improving diet can improve mental health symptoms even in those without pathological malnutrition. This speaks to the perpetuating contributions of diet in less extreme circumstances than severe AN. The converse of a perpetuating factor, in many cases, is an intervention that may serve as a *treatment*. This is evident in considering the WD in contrast to the Mediterranean Diet (MD). The MD is high in vegetables, whole grains, legumes, olive oil and low in saturated fats, salt and refined sugars. While the WD is thought of as a precipitating and perpetuating factor in inflammation and poor physical and mental health, the MD may be the opposite - a treatment intervention.

In the Supporting the Modification of lifestyle In Lowered Emotional States (SMILES) randomized controlled trial (RCT), a modified Mediterranean diet (MD) intervention group had greater improvements in depressive symptoms compared to a social support control group, suggesting that dietary improvements may be efficacious and potentially an acceptable treatment for depression (65). In the Healthy Eating for LiFe with a MEDiterranean-style diet (HEFIMED) RCT, a MD intervention supplemented with fish oil, nutrition education, and cooking classes was associated with a reduced ratio of erythrocyte omega-6 FAs to omega-3 FAs and reduced depressive symptoms (66). Additionally, research has shown that making dietary improvements in an outpatient setting during a depressive episode will significantly reduce depression and anxiety symptomatology to both a statistically significant and a clinically relevant extent (67).

Some RCTs have shown associations between ingesting probiotics [live microorganisms that, when administered in adequate amounts, confer a health benefit to the host (68)] and improved mental health symptoms, including in people with major depressive disorder (MDD) (69, 70), multiple sclerosis (71), and healthy older adults (72). In an RCT of participants with MDD, 8-weeks of probiotic (*Lactobacillus helveticus* and *Bifidobacterium longum*) supplementation resulted in a significant decrease in Beck Depression Inventory score compared to placebo and even compared to a prebiotic supplementation (a substrate that is selectively utilized by host microorganisms conferring a health benefit; *galactooligosaccharide*) (69). In another RCT, patients with low-moderate depression treated with probiotics compared to prebiotic or placebo showed increased brain-derived neurotropic factor (BDNF) levels and improved depressive symptoms (70). In the RCT involving patients with multiple sclerosis, multi-strain probiotic supplementation over 6 months resulted in a significant increase in BDNF levels, a significant reduction in IL-6 levels, as well as significant improvements in depression scores (71). On a larger scale, a recent meta-analysis of small RCTs from the Canadian Network for Mood and Anxiety Treatments Taskforce (CANMAT) has reported early interest in adjunctive probiotic use for depressive symptoms, with *Lactobacillus* and *Bifidobacterium* spp. being the most well-studied (73).

The use of fecal microbiota transplantation (FMT) has also been studied in human subjects as a method of decreasing mental illness symptoms. To validate the GM as a mechanism of perpetuating such symptoms, FMT research has shown promising results, to date. Transplantation from depressed humans into antibiotic-treated mice, for example, leads to anxiety and despair-like behaviors in the mice (74), ostensibly through colonization of select *Bacteroidetes* species (75). Similar models have been used to conduct human-to-mouse FMT from patients with attention deficit hyperactivity disorder (ADHD), which found that the mice developed anxiety behaviors and brain changes compatible with ADHD pathophysiology (76). Interestingly, this has also been demonstrated in the context of patients with schizophrenia with FMT from humans to mice (77).

A human-to-human FMT RCT has demonstrated that FMT from donors with maximal *Lachnospiraceae* and *Ruminococcaceae* was helpful for reducing alcohol use disorder-related events for the 6-month trial in patients with alcohol use disorder and liver cirrhosis (78). Open label trials have also been conducted with human-to-human FMT showing improvement in autism symptoms (79, 80). These results implicate the GM as having a potentially major role in the ongoing pathophysiology of symptoms and thus acting as a perpetuating factor of these mental illnesses (Figure 1C). Such studies pave the way for the further exploration and development of clinically approved means of altering the GM to effectively treat psychiatric illnesses.

Despite this interventional work highlighting the impact of GBM on mental health outcomes, there are barriers to widespread interventions such as probiotic use, FMT or even modifying dietary intake. Dietary modification is currently not a widespread recommendation for patients with a primary mood disorder, anxiety disorder, psychotic disorder, or others. Yet the evidence above suggests that it should be considered as a way of correcting one perpetuating variable, the GBM, of such conditions.

Modifying diet is particularly challenging for EAs as well as adults. Such behavioral changes are influenced by individual motivation, availability/accessibility of specific foods, food knowledge and skills, and social pressures through peer/family influences (81). Overcoming well-established habits is particularly daunting when someone is depressed or anxious (82). Emotional eating is associated with negative emotions, which in turn is associated with indicators of obesity (83). If clinicians are to operationalize diet as a perpetuating factor and use dietary changes to help reduce ongoing mood symptoms, carefully executed interventions are called for with consideration of established behavior change theory. Recognition of the forces and motivators that are unique to EAs, including peer pressure and lack of complete control over food choices, is essential.

### Diet and the microbiome as *protective* factors for mental illness

As mentioned, dietary characteristics have been shown to play an important role in mental wellness. Certain macro and micro-nutrients have evidence for supporting good mental health. The mental health benefits of oral probiotics (defined above), prebiotics [substrates that are selectively utilized by host microorganisms to confer a health benefit (84)], synbiotics (combined probiotics and prebiotics), and postbiotics [preparations of inanimate microorganisms and/or their components that confer a health benefit to the host (85)], may all be protective factors for mental wellness (Figure 1C) (86). Prebiotics include fermented foods, fruits and vegetables and some of their specific subgroups including berries, citrus fruit, and green leafy vegetables (40). Certain nutrients and prebiotics considered beneficial for maintaining mental wellness are also more abundant in the MD (66). Probiotics and synbiotics are considered beneficial to the GM and GBM alike (20, 51, 70, 87, 88). The combination of a prebiotic and postbiotic was found to provide increased benefit compared to either alone in animal models (89). Avoidance of exposure to UPFs and environmental agents that impact the GM are also considered protective for good mental health (43) (Figure 1C).

#### The Mediterranean diet

The MD, which consists of high proportions of fresh fruits and vegetables, dietary fibers, is low in salt and sugars, and is sometimes supplemented with fish oil (66), has been extensively studied in the literature. These factors have been demonstrated to have beneficial effects in the primary and secondary prevention of multiple diseases including type 2 diabetes, various cancers, and in mental illnesses, among others (90). Alterations in the GM, such as increased *bifidobacterial* counts and increased selected SCFAs (91), along with the neuroprotective effects of antioxidant, anti-inflammatory, and increased omega-3PUFA levels seen in the MD, have been associated with positive neurocognitive effects (66). Importantly, however, we acknowledge that the practical costs of following a healthy eating pattern such as a MD must also be considered when making recommendations for patients (92).

Importantly, the MD has been evaluated by Bayes et al. in the context of young adult mental health of males in a controlled trial and found to be beneficial for depression. This study involved dietary planning with a clinical dietitian as well as education about improving dietary intake to reduce processed foods and increase dietary components consistent with the MD. With education and individual planning and support alone, without the researchers controlling food intake, results showed significant improvement in the intervention group (93).

#### Fermented foods

Fermented foods are not a single food type and are defined as foods made through desired microbial growth and enzymatic conversions of food components (94). They can include yogurt, cheese, kefir, kombucha, kimchi, and sauerkraut, for example. Research supports a general consensus that the broad category of fermented food supports good mental health (95). Fermentation of food substances produces multiple beneficial outcomes: functional microorganisms, substances that enhance the proliferation of beneficial bacteria in the gut, and fermented food metabolites that are functionally active (i.e., postbiotics) (96). Fermentation process transforms raw food ingredients through microbial metabolic processes. The resulting products influence microbial composition and function, macronutrient breakdown and absorption, and gut permeability (97), as well as stimulating immune cells and reducing inflammation in the gut (96). For instance, Wastyk et al. (98) found that increasing dietary consumption of fermented foods increased gut microbiota diversity during a 10-week study, which is considered protective. These researchers also noted that measures of inflammatory markers were reduced within the fermented food group, suggesting a possible role for systemic inflammatory reduction from fermented food supplementation (98). Together, these findings suggest that fermented food consumption may be an opportunity for preventing and reducing systemic inflammation that may lead to depressive symptoms (51, 54, 98).

Casertano et al. (99), in their article on preserving mental health, discussed how those fermented foods that contain live microorganisms can reduce dysbiosis, induce healthier GM composition and decrease neuropsychiatric symptoms such as impaired cognition, sleep, depression and anxiety. For example, lactic acid bacteria such as *S. thermophilus*, *L. brevis, L. paracasei, L. fitsaii, L. plantarum*, and *B. adolescentis* produced in some fermented foods are associated with increased bioavailability of gamma-aminobutyric acid (GABA), which regulates stress, anxiety, and depressive-like behavior (99).

Hilimire et al. (100), found that the consumption of fermented foods was negatively associated with social anxiety symptoms in young adults in their cross-sectional study. They hypothesized that those with a higher genetic risk for social anxiety disorder, indexed by high neuroticism, showed fewer social anxiety symptoms when they consumed more fermented foods (100). This suggests a possible nutrigenetic protective factor, though research in this area as it relates to mental health outcomes is sparse.

A dietary intervention study of a non-clinical sample, using GM healthy (whole grains, prebiotic fruits and vegetables, legumes) and fermented foods with reduced intake of sweets and fast food/sugary drinks over 4-weeks, demonstrated a reduction in perceived stress scores with subtle changes in microbial composition and function compared to a control group (101). The perceived stress scores were reduced with greater adherence to the diet, but the effects diminished post-intervention (101).

For some, consuming certain foods like fermented foods (or the MD) to promote health may be viewed as one of the “fad diets,” particularly when claims are juxtaposed with commercial products (102). Yet fermented foods have been part of many cultures historically, and are seeing renewed interest generally (103). The increasing trend among consumption of commercially available kefir and kombucha highlights a potential area for targeting popularization of fermented foods among EAs. However, the accuracy of claims made by commercially available product options has recently been questioned. For example, Metras et al. (104) examined five available kefir products using a 14-day growth period and showed a wide variance in bacteria species not accounted for in the nutrition labels. Further accuracy and quality assurance among labels would support regulatory bodies and researchers in identifying appropriate product recommendations for specific populations. Given that within the same fermented food category products can vary in microbial composition and other important nutritional factors, including added sugars, generalized health claims are difficult to endorse.

#### Future of microbial therapeutics

The mental health benefits of oral probiotics (68), prebiotics (84), synbiotics and postbiotics (85) have been discussed above (and shown in Figure 1C). Studies of the GBM and the changes associated with these entities as protective factors for mental health of humans is still in its early stages, and studies differ in research methods and samples with a variety of confounders and study limitations, such as predominantly involving cross-sectional designs (105).

Studies of the keystone bacteria, *Akkermansia muciniphila* (*AM*), have shown emerging evidence for host metabolic functions and immune responses (106). *AM* is suggested to play an important role in neuropsychiatric diseases such as depression, anxiety and autism spectrum disorder (106) Preclinical studies have shown decreased abundance of *AM* in models of depression and anxiety (106) *AM* treatment improved chronic stress-induced depressive behavior in mice via the regulation of GM and metabolites (107, 108). In human models, abundance of *AM* was negatively correlated in infants of mothers with prenatal psychological distress (109), and human studies are underway to determine the effect of *AM* on GM, the GBM and psychological health. These studies implicate *AM* as a protective factor for good mental health. Our group’s clinical trial (#NCT05022524) is presently ongoing to explore the effect of *AM* in EAs who experienced weight gain from psychotropic medications. We aim to evaluate *AM’s* relative abundance, metabolic profile, weight changes, and changes in mood/anxiety symptoms following use of a prebiotic to facilitate *AM* growth. Such studies emphasize the potential applicability of GM manipulation as either a potential protective measure or treatment for this common side effect of psychiatric medications.

Furthermore, there are a variety of ongoing clinical trials investigating the interconnection between the gut microbiome and mental health. For instance, one planned trial is investigating the relationships between the GM, metabolic factors, diet, and intestinal permeability among children and adolescents (#NCT04330703). Other studies are looking at supplementation across a variety of populations, for instance use of fish oil, probiotic, prebiotic, or diet quality interventions for perinatal mood and anxiety (#NCT06074250), and triglyceride supplementation for adults with low mood (#NCT06058364). There are ongoing studies investigating dietary interventions on mental health, such as the effects of MD interventions (#NCT05927376) and diets rich in fermented food (#NCT06020703) on adult mental health.

Järbrink-Sehgal et al. (105) highlight the need for dedicated longitudinal studies with extended follow-up periods and further investigation utilizing mucosal biopsy and brain imaging to evaluate the mechanisms of GBM communications. Long-term studies are needed to better understand the influence of nutritional interventions such as the MD, prebiotics (including fermented foods), probiotics, synbiotics, postbiotics, and FMT on long-term mental health outcomes and GM changes in EAs, and the extent to which these can operate within the 4P factors for mental health.

## Discussion

This review has explored the use of the “4Ps” for considering interactions between the GBM axis and mental wellbeing. We have outlined factors that are likely to predispose, precipitate, perpetuate and protect against mental illness through the GBM. Hundreds of studies and scientific papers confirm the role of a healthy versus unhealthy diet on good health outcomes at all stages of the life cycle. However, the delivery of this information in the context of mental health care, particularly for EAs, who are still in the formative years of adult food choices and dietary exposures, has been largely absent.

Early child nutrition as well as nutrition during the vulnerable period of emerging adulthood are intervals when food plays a vital role in predisposing toward either a healthy and resilient GM or one that makes the individual vulnerable to disease. Some predisposing factors are not modifiable by the time of contact with a mental health care professional, like genetics, characteristics of birth, infant feeding, or childhood dietary intake. This calls for more research and public health intervention around improving the GM of infants and children, including potential use of prebiotics and probiotics (110).

Precipitating and perpetuating factors can be modifiable. Poor quality diets like those in the current WD, with UPFs as well as high sugar and salt and low fiber, precipitate and perpetuate dysbiosis of the GM (41, 51). Healthy diets like the MD and consumption of fermented foods can promote healthy bacteria and improve function of the GM as protective (66, 91, 99). Addressing systemic inflammatory and GBM-axis related hormonal changes associated with poor diet and high processed food intake through increasing consumption of fermented foods (51, 54, 98), probiotics, prebiotics, synbiotics, postbiotics (72, 88, 111), and promoting a healthy shift in diet (66), are ways to target negative precipitating and perpetuating effects of diet on mental health and the GM. The possibilities of modifying mental illnesses through the use of FMT are exciting and await more research and clinical implementation to be feasible. Eliminating chemicals that disrupt the GM from foods and the environment are also important public health interventions to reduce the onset and chronicity of ailments impacted by chronic inflammation and related diseases, including mental illnesses (43).

Changing or modifying nutritional intake at the level of the individual is notoriously difficult and requires a personalized and flexible approach to meet the individuals’ socioeconomic and cultural preferences, among other factors. Increased work demands, time constraints, lack of motivation and financial cost have been reported to be barriers toward healthy eating behaviors among EAs (112, 113). Advertisements for fast-food products attempt to benefit from these barriers. EAs were shown to easily recall energy-dense and nutrient-poor foods compared to healthier food alternatives and perceive the former as more affordable and convenient compared to healthy alternatives (114). Shifting policies toward increased advertisement of healthy foods and targeting cost and accessibility of healthy food choices are opportunities to promote healthy eating (114–116).

Increasing understanding around healthy eating and benefits of healthy food among young adults may be helpful, given that food literacy has been shown to be positively associated with healthy eating behaviors, suggesting that programs tailored to food literacy among EAs may be an opportunity for increasing healthy eating practices (15). With this in mind, increasing access to registered dietitians (RDs) for EAs with mental illness can help with improving dietary habits. For EAs in particular, assessing the use of social media to engage with RDs may be an important area of research and evaluation. Increasing the availability of nutrition education at the elementary school level has the potential for long-lasting and habit-forming healthy dietary changes (117) that will more likely be cost-effective to the healthcare system and the economy compared with the long-term health care consequences of a poor diet. Cost–benefit analyses are recommended to investigate this.

Importantly, despite the emerging evidence, information about probiotics, prebiotics or fermented foods are absent from the Canadian and American food/dietary guidelines. The International Scientific Association for Probiotics and Prebiotics (ISAPP) continues to advocate for probiotic, prebiotic, and fermented food recommendations to consumers, clinicians, scientists, policy makers and public health associates (118, 119).

While intervention at the individual level is important and, from the perspective of a clinician, the only way to promote the 4Ps to patients in the role of improving the GM for mental health benefits, the role for public health cannot be underestimated. Dietary guidelines to help direct individual food choices are helpful public health interventions. Challenges to GM health and resilience from additives into the food chain and environmental exposures to chemical compounds that impact the GM need consideration, and may play a pivotal role in predisposing and perpetuating GBM disruption and mental ill health in the population at large (43, 44, 120, 121).

The period of emerging adulthood is an epoch of change wherein the predisposing and protective factors contributing to GBM status in earlier life are strongly influential for mental health. A variety of choices and involuntary exposures then create precipitating and/or perpetuating factors that contribute to the onset of either mental illness or resilience. We emphasize the need for focused studies/interventions in youth and EAs as an opportunity to understand this critical period within the context created by the 4Ps and in a way that is useful for clinicians and patients alike for creating long-lasting positive impacts.

## Author contributions

MWar: Data curation, Investigation, Validation, Writing – review & editing. CO’C: Conceptualization, Data curation, Investigation, Supervision, Validation, Writing – original draft, Writing – review & editing. JL: Conceptualization, Data curation, Validation, Writing – original draft. JB: Conceptualization, Data curation, Investigation, Supervision, Validation, Writing – original draft, Writing – review & editing. DW: Conceptualization, Data curation, Investigation, Supervision, Validation, Writing – original draft, Writing – review & editing. JK: Conceptualization, Data curation, Validation, Writing – original draft, Writing – review & editing. MWam: Validation, Writing – review & editing. EO: Conceptualization, Data curation, Investigation, Project administration, Supervision, Validation, Writing – original draft, Writing – review & editing.
